# Pennsylvania Hospital

**Published:** 1849-03

**Authors:** Spenser Sergeant

**Affiliations:** Resident Physician; Pennsylvania Hospital


### Pennsylvania Hospital.—*Surgical Wards.—Service of* Dr. Norris.

*Cases admitted since January 15th*, 1849.

Abscess 1, Burns 2, Chilblains 2, Concussion (brain) 1, Fractures, simple, 12; viz.: Nose 1, Lower Maxilla 1, Humerus 2, Forearm 2, Femur 2, Patella 1, Leg 3; Compound 4; viz.: Fingers 1, Forearm 1, Leg 2; Gonorrhœa 1, Hemorrhoids 1, Hernia 1, Hydrocele 1, Inflamed hand 1, Inflamed leg 1, Necrosis (femur) 1, Ophthalmia (purulent) 1, Onychia 1, Orchitis 2, Paronychia 1, Retention of Urine 1, Syphilis 7, Stricture of Urethra 1, Ulcer 6, Wounds 18; viz.: Contused 8, Incised 3, Lacerated 2, Punctured 2, Gun shot 3.

*Discharged since Jan. 15th, 1849.*

| | Cured. | Relieved. | By request. | Died. |
|---|---|---|---|---|
| Abscess, - - - - - - | 2 | 0 | 0 | 1 |
| Burn, - - - - - - - | 0 | 0 | 0 | 1 |
| Fractures, simple, viz. : Thigh 2, Leg 3, Fore-arm 1, Arm 1, - - - | 7 | 0 | 0 | 0 |
| Fractures, compound, viz. : Upper Maxilla 1, Fingers 1, Leg* 1, Hand† 1, | 4 | 0 | 1 | 0 |
| Gonorrhœa, - - - - - | 1 | 0 | 1 | 0 |
| Hernia, - - - - - - | 0 | 1 | 0 | 0 |
| Hydrocele, - - - - - | 0 | 1 | 0 | 0 |
| Inflamed hand, - - - - | 3 | 0 | 0 | 0 |
| " arm, - - - - - | 1 | 0 | 0 | 0 |
| " knee-joint,‡ - - - | 0 | 0 | 0 | 1 |
| Luxation, - - - - - | 1 | 0 | 0 | 0 |
| Necrosis, - - - - - | 0 | 0 | 1 | 0 |
| Orchitis, - - - - - | 1 | 0 | 0 | 0 |
| Paronychia, - - - - - | 2 | 0 | 0 | 0 |
| Polypus of nose, - - - - | 0 | 1 | 0 | 0 |
| Syphilis, - - - - - | 9 | 0 | 0 | 0 |
| Sprain, - - - - - - | 1 | 0 | 0 | 0 |
| Ulcer, - - - - - - | 5 | 0 | 2 | 0 |
| Wounds 16, viz. : | | | | |
| Contused, - - - - - | 8 | 0 | 0 | 0 |
| Lacerated, - - - - - | 0 | 0 | 0 | 1 |
| Punctured, - - - - - | 3 | 0 | 0 | 0 |
| Incised, - - - - - - | 1 | 0 | 1 | 0 |
| Gunshot, - - - - - - | 2 | 0 | 0 | 0 |
| | 51 | 3 | 5 | 4 |

The fatal case under the head of Abscess was an excellent example of the purulent or suppurative diathesis. The man was about 30 years of age, by trade a tailor, and up to this time healthy. His parents, as well as his brothers and sisters, have good constitutions. He had been in the habit of drinking, and sometimes to excess. He had syphilis about five years before. He was originally admitted into the Medical Ward for erythema. His whole body, face and limbs, were covered with a pale blush, resembling subacute erysipelas, and his right foot was inflamed and swollen. He stated that he had hurt his foot about ten days before admission, but had continued at his work till within two or three days, when the erythema appearing he applied to the hospital. The constitutional disturbance was great. His pulse over 100 and feeble;

* Cured by amputation, double flap operation of forearm.

† Cured by amputation, circular incision of thigh.

‡ A young man of marked scrofulous diathesis. Inflammation went rapidly on to suppuration and ulceration, with such severe constitutional disturbance as to put amputation out of the question.

his tongue coated, &c. The erythema disappeared in a day or two, but his foot beccame worse, and soon showed signs of suppuration. The pus was discharged first from an ulcerated opening between the fourth and fifth toes, and afterwards counter-openings were made at different points on the back and sole of his foot; very soon a large abscess was discovered on the right side, extending from near the lower point of the scapula to the sternum. This was opened, and five or six ounces of healthy pus discharged. From this time abscesses appeared successively at different parts of his body and limbs. They were of different sizes, and were preceded by no inflammatory redness, pain or tenderness on pressure. The collections were nearly all encysted, and after being punctured the sacs contracted and the discharge ceased. In some, however, there was no disposition in the sac to contract, and the discharge continued thin, dark, and very offensive. Towards the close of his illness the abscesses formed in greater number and with greater rapidity; so that at his death no fewer than eighteen purulent collections were observed on the surface of his body. He was sustained by the most stimulating and nourishing articles, but the discharge and great constitutional irritation gradually wore him out.

The autopsy was made 13 hours after death. Emaciation very great; rigidity incomplete; eighteen abscesses were observed on the surface of the body, and two were found on the inner face of the sheath of the rectus abdominis muscle. Thorax, recent adhesions on each side. Lungs pale in front, violet-coloured posteriorly. On cutting into the right lung numerous points of pus not contained in any cavity were observed. On the anterior face of the left lung, hard points of a dark colour were felt under the pleura, which, when cut into, were found to be minute cavities enclosing pus. Posteriorly the lung was infiltrated throughout with pus. Liver enormously enlarged, of a pale yellow colour. Spleen, good consistence, light colour, medium size; no purulent deposits were found either in liver or spleen. Kidneys, medium size, pale, containing in tubular portions numerous small collections of pus. Stomach and small intestines healthy.

The death from lacerated wound of the finger illustrates the fatal results which sometimes follow slight injuries. Owen Nevins, aged 48, applied for admission Jan. 13th, 1849, with a lacerated wound of the little finger of the right hand, caused by its being caught between two barrels. He was put to bed, the hand and arm elevated on a splint and cold dressings applied. Inflammation, attended by a great deal of œdema, but with little heat or redness, extended up the hand and arm, and the wound presented a sloughy appearance. Suppuration was first detected on the back of the hand, and a free opening was made to discharge the pus. Counter-openings were made in the palm and afterwards in the arm. In the meantime his general symptoms became very threatening; his pulse was feeble and frequent, his tongue dry and brown, and although tonics and stimuli were freely given, his condition was not improved. The punctures made to evacuate the pus ulcerated, and sloughing of the cellular tissue took place. Shortly after he had three attacks of arterial hemorrhage from the ulcerated openings in the arm, in each of which he lost a considerable amount of blood. It was deemed improper to amputate in his enfeebled condition. He gradually sank and died, Feb. 3d, 1849.

Upon examination after death, all the organs were found pale, but healthy, with the exception of the spleen. The spleen was enlarged, softened down to a reddish pulp, and contained numerous deposits of pus. Throughout the abdomen there were traces of old peritoneal inflammation, the small intestines bound together, and the peritoneum opaque and thickened.

SPENSER SERGEANT, M. D.,
Resident Physician.

Pennsylvania Hospital, Jan. 16th, 1849.

